# Gene order alignment on trees with multiOrthoAlign

**DOI:** 10.1186/1471-2164-15-S6-S5

**Published:** 2014-10-17

**Authors:** Billel Benzaid, Nadia El-Mabrouk

**Affiliations:** 1 Département d'Informatique (DIRO), Université de Montréal, Montréal, H3C3J7 Québec, Canada

## Abstract

We relate the comparison of gene orders to an alignment problem. Our evolutionary model accounts for both rearrangement and content-modifying events. We present a heuristic based on dynamic programming for the inference of the median of three genomes and apply it in a phylogenetic framework. multiOrthoAlign is shown accurate on simulated and real datasets, and shown to significantly improve the running-time of DupLoCut, an "almost" exact algorithm based on linear programming, developed recently for the same problem.

## Introduction

A major requirement in comparative genomics is to be able to compare genomes based on their whole content. This is necessary for a myriad of applications such as phylogenetic reconstruction, orthology and paralogy identification, ancestral reconstruction and the study of evolutionary events. Consequently, a large variety of algorithms have been developed for the comparison of whole-genome sequences with partial or no information on gene annotation. Most of them are based on first identifying, in a pair-wise alignment dotplot, local alignments (anchors, syntenies) with high similarity score, and then chaining them in a way maximizing an alignment score (cf. e.g. MUMmer [1], BLASTZ [2], LAGAN [3], DAGchainer [4], progressiveMauve [5]). Similarity scores are computed according to the local mutations (nucleotide substitutions and indels) inferred from the alignment. Other approaches compare genomes in terms of building block organization. Although a recently developed method does not require any preliminary information on gene families [6], most of them assume a full or partial annotation of genomes, or a previously established large coverage of genomes in terms of syntenic blocks. Given two genomes represented as ordered sequences of genes (or building blocks), the rearrangement approach consists in finding a sequence of global evolutionary events transforming one gene order to the other. Early work on genome rearrangement focused on sorting permutations (no duplicates) by rearrangements (inversions, translocations, transpositions) [7-9]. More recently, a variety of studies have considered the more difficult case of genomes with duplicates evolving through rearrangements, but also through content modifying operations such as duplications and losses (reviews in [10,11]). Other model-free approaches based on conserved synteny, with no assumption on the evolutionary mechanisms, have also been developed [6,12-16].

In a recent set of papers [17-19] we related the comparison of two gene orders to an alignment problem: find an alignment between the two gene orders that can be interpreted by a minimum number of evolutionary events (rearrangements and content-modifying operations). Although alignments are *a priori *simpler to handle than rearrangements, this problem has been shown NP-hard for the duplication-loss model of evolution [17,18,20]. Exact exponential-time algorithms based on linear programming [19,20] and a polynomial-time heuristic based on dynamic programming [17] have been developed for this model. Recently [21], we developed OrthoAlign (alignment of orthologs), a time-efficient heuristic for the gene order alignment problem, that extends the dynamic programming approach to a model including rearrangements (inversions and transpositions).

Sequence and gene order alignments are useful for ancestral inference purposes. As explained in [19], a "labeled" pair-wise gene order alignment can be translated into a common ancestor and an evolutionary scenario leading to the two compared gene orders. Such an alignment approach for ancestral inference is relevant if the two gene orders reflect enough conservation so that we can assume that only few events have occurred since the divergence of the lowest common ancestor of the two genomes. For such closely related species, events can be assumed to be non-overlapping (each gene involved in at most one event) and thus still visible in the alignment. The gene-order alignment approach has been shown useful to decipher the evolutionary mechanisms that have shaped the tRNA gene repertoires of the bacterium *Bacillus *[19].

Here, we undertake the next step, which is using the alignment approach on a phylogeny: infer ancestral genomes identified with each speciation node of a phylogenetic tree. The alignment on a tree problem introduced by Sankoff *et al*. in [22], consists in finding assignments of internal nodes in a way minimizing the total branch length of the tree according to a given distance. The result is, not only a set of ancestral genomes, but also a multiple alignment for extant sequences. As trying all possibilities for internal node assignments is intractable, iterative heuristics on subtrees are usually considered, the most popular being the median-based heuristic [10,23]: (1) find an initial assignment for internal nodes; (2) in a post-order traverse of the tree, improve the assignment of each internal node *u *by considering the median of the leaf-assignments of the 3-star tree centered on *u*, i.e., the tree formed by the three neighbouring nodes of *u*; (3) repeat until no improvement on the tree distance can be made. In the case of genomes represented as gene orders, applying the exact 2-SPP (2-Small Phylogeny Problem) algorithm [19] or OrthoAlign [21] to the cherries of the phylogeny can be used for an initial assignment. As for the iterative step, an efficient algorithm for the median problem has to be found. Although NP-hard for most versions of the problem [24-26], efficient heuristics have been developed for various nucleotide and rearrangement distances. As for the duplication-loss model of evolution, DupLoCut, an "almost" exact algorithm based on linear programming has been presented in [20].

In this paper, we present multiOrthoAlign for the alignment of a set of gene orders related through a phylogenetic tree. It is based on a dynamic programming approach generalizing OrthoAlign [17,21] to a 3-star tree, under a model involving a wide range of evolutionary events. multiOrthoAlign is compared with DupLoCut [20], the most closely related algorithm. Experiments on simulated and real datasets reveal similar accuracy for both algorithms, but with a significant improvement in running time for multiOrthoAlign.

## Method

We consider uni-chromosomal genomes represented as strings of signed characters from an alphabet Σ, where each character represents a gene family. Each character may appear many times in a genome *G*, all such positions corresponding to genes belonging to the given gene family. The sign of a gene represents its transcriptional orientation. Let *X *= *x*_1_*x*_2 _*· · · x_n _*be a string. We call the *reverse *of *X *the string *−X *= *−x_n _· · · *− *x*_2 _− *x*_1_. We denote by *X*[*i, i *+ *k*] the *substring *of *X *formed by the consecutive genes of the interval [*i, i *+ *k*].

A *phylogeny *or *species tree * S for a set Γ of genomes is a tree with a one-to-one correspondence between the leaves of  S and the species of Γ, reflecting the evolution of the genomes through speciation. Although the method developed in this paper does not require any assumption on the species tree, for ease of presentation, we consider binary and rooted phylogenies. An internal node of  S corresponds to a speciation event and an assignment for that node corresponds to the genome at the moment of speciation. A *phylogenetic alignment * S for  S is the tree  S augmented with an assignment of one string for each internal node of  S. When no ambiguity, we will make no difference between a node and its assignment. Two nodes are *related *if they belong to the same path from a leaf of  S to the root, and *unrelated *otherwise. For two related nodes *A *≠ *X, A *is an *ancestor *of *X *if *A *is closer to the root of  S than *X*. For two unrelated nodes *X *≠ *Y*, they are *siblings *if they share the same parental node. A pair of siblings is called a *cherry*. Moreover, we call a *3-star *of  S and we denote by *A|XY *a star-tree with three leaves *A, X, Y *such that *X *and *Y *are two siblings in  S and *A *is the immediate ancestor of the parent *M *of *X *and *Y. M *is called the center of *A|XY*.

### The evolutionary model

We assume that present-days genomes have evolved from an ancestral genome through rearrangement and content-modifying events, each event (operation) acting on a uni-chromosomal genome *X *and leading to a new uni-chromosomal genome *Y*. An operation is denoted by *O*(*k*) = (*O^S^, O^T^*), where *O *is the operation type, *k *is the length of the operation, *O^S ^*is the *source*, i.e., the substring affected by the event and *O^T ^*is the *target*, i.e., the new substring resulting from the event. Characters of *O^S ^*and *O^T ^*are said to be *covered *by the operation. The mostly considered content-modifying operations are duplications and losses, where:

• A **Duplication ***D*(*k*) = (*D^S ^*= *X*[*i, i *+ *k *− 1], *D^T ^*= *Y *[*j, j *+ *k *− 1]), where *Y*[*j, j *+ *k *− 1] = *X*[*i, i *+ *k *− 1], is an operation that copies the substring *X*[*i, i *+ *k *− 1] of size *k *to a location *j *outside the interval [*i, i *+ *k *− 1] (i.e. preceding *i *or following *i *+ *k *− 1);

• A **Loss ***L*(*k*) = (*X*[*i, i *+ *k *− 1], ∅) (∅ for empty string) is an operation that removes the substring *X*[*i, i *+ *k *− 1] from genome *X*.

The mostly considered uni-chromosomal rearrangements are reversals and transpositions, where:

• A **Reversal **(or inversion) *R*(*k*) = (*X*[*i, i *+ *k *− 1], *Y *[*i, i *+ *k *− 1]), where *Y *[*i, i *+ *k *− 1] = *−X*[*i, i *+ *k *− 1], is an operation that transforms the substring *X*[*i, i *+ *k *− 1] into its reverse;

• A **Transposition ***T*(*k*) = (*X*[*i, i *+ *k *− 1], *Y *[*j, j *+ *k *− 1]), where *Y*[*j, j *+ *k *− 1] = *X*[*i, i *+ *k *− 1], is an operation that moves the substring *X*[*i, i *+ *k *− 1] to another position *j *outside the interval [*i, i *+ *k *− 1].

Denote by  O the set of operation types. We will describe our approach for O={D,L,R,T}. Including other events, such as inverted duplications or inverted transpositions with target being the reverse of the source, insertions which are the counterparts of losses, or substitutions replacing a string with another of the same size, do not add any complexity to the problem. Notice however that the more operations we include to the model, the more challenging is the problem of assigning appropriate operations costs.

Let  S be a phylogeny and *X, Y *be two nodes of  S. If *X *and *Y *are related, say *X *is an ancestor of *Y*, then a *history O*_*X*→*Y *_for *X *and *Y *is a sequence of events (possibly of length 0) transforming *X *into *Y*. Otherwise, if *X *and *Y *are unrelated, then a *history *for *X *and *Y *is a triplet (*A, O*_*A*→*X*_, *O*_*A*→*Y*_), where *A *is an assignment of the lowest-common ancestral node of *X *and *Y*. We call a *visible history for X and Y *a history where the source and target of each operation is a substring of *X *or *Y*.

Finally, let *A|XY *be a 3-star of  S. A history for *A|XY *is a quadruplet (*M, O*_*A*→*M*_, *O_M _*_→*X*_, *O_M _*_→*Y*_) where *M *is an assignment of the center of the 3-star. A *visible history for A|XY *is a history where the source and target of each operation is a substring of *A, X *or *Y*.

Notice that a duplication with source and target in two different genomes can be interpreted as a duplication followed by the loss of the source (a relaxation of visibility), or alternatively as a transposition, or even as a horizontal gene transfer between the two considered genomes. We will take this general view of a duplication, which implicitly integrates transpositions.

### Genome alignment

We begin by recalling the classical notion of an alignment of strings (genomes) Γ = {*X_k _*: 1 *≤ k ≤ γ*}. Let Σ*^− ^*= Σ ∪ {*−*} be the alphabet Σ augmented with an additional character '-' called a gap. Then an *alignment *for Γ is a set $\bar{\text{Γ}}=\left\{\bar{X_{k}}:1\leq k\leq \gamma \right\}$ of strings obtained by filling *X_k _*with gaps, such that the resulting *aligned genomes *have equal length *λ*, and for each position *i*, 1 *≤ i ≤ λ*, the column *i *is *not empty *in the sense that at least one of $\bar{X_{k}}\left[i\right]$, for 1 *≤ k ≤ γ*, is not a gap. The *induced alignment *for a subset Γ' ⊂ Γ is the alignment Γ' obtained by removing from $\bar{\text{Γ}}$ all genomes that are not in Γ' and all empty columns. Given a pair $\left(\bar{X_{l}}\left[i\right],\bar{X_{m}}\left[i\right]\right)$ of aligned characters, it is a *match *if $\bar{X_{l}}\left[i\right]=\bar{X_{m}}\left[i\right]\in \text{Σ}$, a *mismatch *if $\bar{X_{l}}\left[i\right]\neq \bar{X_{m}}\left[i\right]$ both being in Σ and a *gap *if $\bar{X_{l}}\left[i\right]\in \text{Σ}$ and $\bar{X_{m}}\left[i\right]{=}^{\prime }{-}^{\prime }$.

A multiple alignment is expected to reflect the evolutionary events that have led to the present-day genomes. The notion of an *alignment labeling *has been introduced in [19] for a pair-wise alignment. It relates each column of the alignment to a given operation. Generalization to an arbitrary number of genomes is given bellow. We will make use of this definition later in the context of a 3-star history.

**Definition 1 ***Let *$\bar{\text{Γ}}=\left\{\bar{X_{k}}:1\leq k\leq \gamma \right\}$*be an alignment of length λ. A labeling *$L\left(\bar{\text{Γ}}\right)$ for $\bar{\text{Γ}}$*is a set of operations covering the characters of the given sequences. For any l and m in *[1, *γ*] *with l *≠ *m and any i*, 1 *≤ i ≤ λ, such that $Xl\left[i\right]\neq^{\prime }{-}^{\prime }$*,

(*X_l_*[*i*], *X_m_*[*i*]) *is covered by at most one operation of $\bar{\text{Γ}}$ as follows:*

• *if a match, then it is covered by no operation;*

• *if a mismatch, then it is covered by a reversal;*

• *if a gap, then it is covered by one of the other operations of  O*.

with the restriction that, if the two genomes are related, say X_l _is an ancestor of X_m_, then the source of the operation should be in X_l _and the target should be in X_m_.

A *labeled alignment *is an alignment $\bar{\text{Γ}}$ accompanied with a labeling $L\left(\bar{\text{Γ}}\right)$. We simply refer to a labeled alignment by its labeling $L\left(\bar{\text{Γ}}\right)$. The *cost of a labeled alignment *is the sum of costs of all its labeling events.

The above definition does not ensure a valid interpretation of a labeled alignment in terms of an evolutionary history (*A, O*_*A*→*X*_, *O*_*A*→*Y*_) for two genomes *X *and *Y*. We showed in [19] that a pair-wise labeled alignment is valid if and only if it is free from cycles, where cycles are defined as follows.

**Definition 2 ***Let  O be a set of operations. It induces a *cycle *if there is a permutation O*_1_, *O*_2_, *· · · O_h _of  O events such that the substrings $O_{p}^{T}$ and $O_{p+1}^{S}$ overlap (a suffix of $O_{p}^{T}$ is a prefix of $O_{p+1}^{S}$*), *for each *1 *≤ p ≤ h *− 1, *and the substrings $O_{h}^{T}$ and $O_{1}^{S}$ overlap*.

A *feasible labeled alignment *is a labeled alignment with no cycles. We showed in [19] the one-to-one correspondence between feasible labeled alignments and visible histories for two genomes *X *and *Y *in case of an evolution through duplications and losses.

### Phylogenetic alignment

Let  S be a species tree for a genome set Γ. Call a *feasible labeled phylogenetic alignment *for  S a phylogenetic alignment $\bar{S}$ accompanied with a feasible labeled alignment for each cherry (*X, Y*) of $\bar{S}$, in other words a visible history (*A, O*_*A*→*X*_, *O*_*A*→*Y*_) for each (*X, Y*). Such a feasible labeled phylogenetic alignment leads to a multiple alignment for Γ: traverse $\bar{S}$ in post-order and iteratively incorporate alignments of cherries in a current multiple alignment which is initially empty.

Let *A *and *X *be two genomes of  S with *A *being an ancestor of *X *and let *O*_*A*→*X *_= {*O*_1_(*k*_1_), *· · · O_m_*(*k_m_*)} be a history for *A *and *X*. The cost of *O*_*A*→*X *_is defined as $C\left(O_{A\to X}\right)=\sum_{i=1}^{m}c\left(O_{i}\left(k_{i}\right)\right)$, where *c*(*O_i_*(*k_i_*)) is the cost of the operation *O_i_*(*k_i_*). Let $O_{A\to X}$ be the set of all possible histories transforming *A *into *X*. We define $C\left(A\to X\right)={\text{min}}_{O_{A\to X}\in O_{A\to X}}C\left(O_{A\to X}\right)$. Now, the *phylogenetic alignment problem*, is to infer a feasible labeled phylogenetic alignment for  S minimizing the sum of costs of all branches of  S.

The relaxed phylogenetic alignment problem with no restriction on visibility, i.e. the problem of assigning ancestral configurations leading to a minimum cost for the tree, has been shown to be NP-hard for most formulations in terms of type of genomes and different distances. A classical heuristic strategy is known as the *steinerization approach *[23]. It begins with an initial assignment for the internal nodes of  S, and in a post-order traversal it improves each internal node assignment by solving a 3-star problem defined as follows.

3-star Problem:

INPUT: A 3-star phylogeny *A|XY*.

OUTPUT: A visible history (*M, O*_*A*→*M*_, *O*_*M*→*X*_, *O*_*M*→*Y*_) for *A|XY *minimizing the cost:

$$C\left(A\to M\right)+C\left(M\to X\right)+C\left(M\to Y\right).$$

In the case of symmetrical operations, such as nucleotide substitutions or indels, or gene order rearrangements, the direction of evolution can be ignored, which leads to the median problem: find *M *minimizing *C*(*M, A*) + *C*(*M, X*) + *C*(*M, Y*). However, this is not the case for content-modifying operations, as for example a duplication from *A *to *X *is rather a loss from *X *to *A*, and therefore the evolutionary direction cannot be ignored in this case.

For the evolutionary model of interest, the restriction of the phylogenetic alignment problem to a cherry has been considered in [17,19]. The developed algorithm can be used for the initialization step: traverse the tree in a depth-first manner and compute successive ancestors of pairs of nodes. Here, we extend our study to a 3-star phylogeny, which allows for the application of the aforementioned steinerization approach. Notice that the phylogenetic alignment problem has been shown NP-complete for the duplication-loss model of evolution, already for two species [20,17,18].

The 3-star Problem

**Figure 1:**
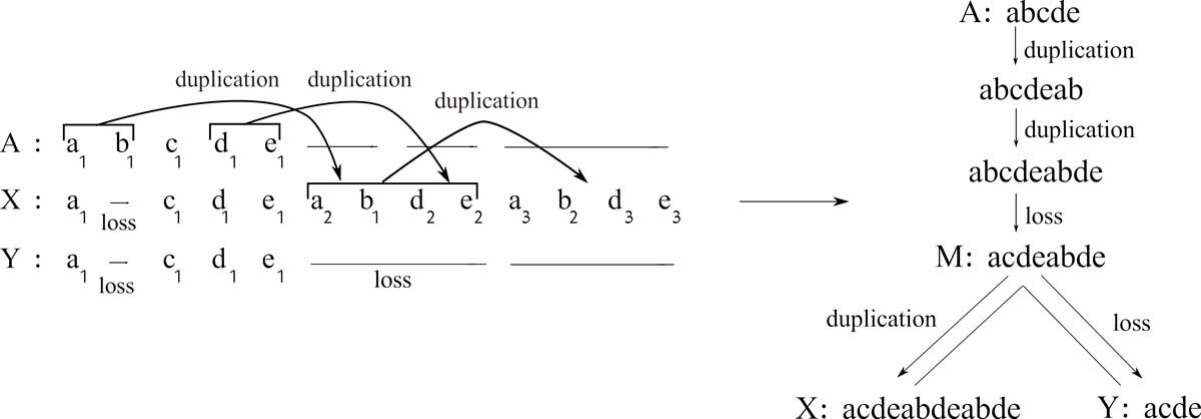
**A labeled alignment for three strings and their visible history**. Left: a labeled alignment for strings *A*="abcde", *X*="acdeabdeabde" and *Y*="acde". Right: The visible history for *A|XY *and the center *M *obtained from this alignment.

We first show that the 3-star problem for a 3-star *A|XY *reduces to finding a feasible labeled alignment for {*A, X, Y*} of minimum cost. It is easy to see that any visible history for *A|XY *leads to a unique feasible labeled alignment for {*A, X, Y*}. Conversely, let $L\left(Ā,\bar{X},Ȳ\right)$ be a feasible labeled alignment for a 3-star *A|XY*. A corresponding visible history for *A|XY *can be obtained as follows (see Figure 1 for an example):

• Define (*M, O*_*M*→*X*_, *O*_*M*→*Y*_) as the visible history corresponding to the induced feasible labeled alignment for *X *and *Y*.

• Consider the alignment $\left(Ā,\bar{M}\right)$, where $\bar{M}$ is the aligned genome *M *corresponding to the above history.

• Define $L\left(Ā,\bar{M}\right)$ as follows. For each *i *such that $\left(Ā\left[i\right],\bar{M}\left[i\right]\right)$ is not a match:

- If $\bar{X}\left[i\right]=Ȳ\left[i\right]$ then include in $L\left(Ā,\bar{M}\right)$ the operation of $L\left(Ā,\bar{X},Ȳ\right)$ covering the column $\left(Ā\left[i\right],\bar{X}\left[i\right]\right)$ (or alternatively (Ā[i],Ȳ[i])).

- Otherwise $\bar{M}\left[i\right]$ should be equal to $\bar{X}\left[i\right]$ or Ȳ[i]. Assume w.l.o.g. that $\bar{M}\left[i\right]=\bar{X}\left[i\right]$. Then include in $L\left(Ā,\bar{M}\right)$ the operation of $L\left(Ā,\bar{X},Ȳ\right)$ covering the column $\left(Ā\left[i\right],\bar{X}\left[i\right]\right)$.

Therefore, given a 3-star *A|XY*, we focus here on the problem of finding a feasible labeled alignment for {*A, X, Y*} of minimum cost.

Let *C*(*i, j, k*) (*C^f^*(*i, j, k*) respectively) be the minimum cost of a labeled (feasible labeled respectively) alignment of three prefixes *A*[1, *i*], *X*[1, *j*] and *Y *[1, *k*] of *A, X *and *Y*, for all 1 *≤ i ≤ |A|*, 1 *≤ j ≤ |X| *and 1 *≤ k ≤ |Y |*. **Step 1 **described bellow gives a heuristic for computing *C*(*i, j, k*) and **Step 2 **a heuristic for computing

$$C^{f}\left(\left|A|,|X|,|Y\right|\right)\text{ from }C\left(\left|A|,|X|,|Y\right|\right).$$

• STEP 1. FINDING A LABELED ALIGNMENT BY A DYNAMIC PROGRAMMING APPROACH.

As explained previously, transpositions are implicitly considered by allowing the source and target of a duplication to belong to two different genomes. Therefore,

we will restrict our presentation to the model O={D,L,R}.

To compute *C*(*i, j, k*), we consider all the possibilities for the last column of an alignment of the three prefixes *A*[1, *i*], *X*[1, *j*] and *Y*[1, *k*] and interpret it by the minimum number of operations. In the following, a column is represented as a triplet of characters from Σ*^−^*, were different letters denote different characters of Σ. Clearly, each column can be interpreted by no more than 2 operations. If two operations are required to interpret a given column, then we assume them to be of the same size. This eliminates the case of a column of the form [*a, x, y*], as this would require two reversals of different sizes.

*C*(*i, j, k*) is the minimum over all the computed costs.

1 [*a, a, a*]: All matches.

$$M\left(i,j,k\right)=\left\{\begin{array}{cc} C\left[i-1,j-1,k-1\right]\; & \text{if}\;A\left[i\right]=X\left[j\right]=Y\left[k\right]\\ +\infty \;\;\;\;\;\;\; & \text{otherwise} \end{array}\right)$$

2 [*a, x, x*]: Reversal in both *X *and *Y *(i.e. in *M*).

$$R_{XY}\left(i,j,k\right)=\left(\begin{array}{cc} min_{m\in E}\left(C\left[i-m,j-m,k-m\right]+c\left(R\left(m\right)\right)\right)\; & \text{if}\;E\neq \emptyset \\ +\infty & \text{otherwise} \end{array}\right)$$

where *E *is the set {*e*_1_, *e*_2_, . . . , *e_l_*} of maximum cardinality such that *A*[*i−e_p_, i*] is the reverse of both *X*[*j *− *e_p_, j*] and *Y *[*k *− *e_p_, k*] for all 1 *≤ p ≤ l*.

3 [*a, x, a*]: Reversal in *X*. (The case [*a, a, y*] is treated similarly)

$$R_{X}\left(i,j,k\right)=\left(\begin{array}{cc} min_{m\in E}\left(C\left[i-m,j-m,k-m\right]+c\left(R\left(m\right)\right)\right) & \text{if}\;E\neq \emptyset \\ +\infty & \text{otherwise} \end{array}\right)$$

where *E *is the set {*e*_1_, *e*_2_, . . . , *e_l_*} of maximum cardinality such that *A*[*i *− *e_p_, i*] = *Y *[*k *− *e_p_, k*] and *A*[*i−e_p_, i*] is the reverse of *X*[*j *− *e_p_, j*] for all 1 *≤ p ≤ l*.

4 [*−, x, x*]: Duplication in both *X *and *Y *(i.e. in *M*)

$$D_{XY}\left(i,j,k\right)=\left(\begin{array}{cc} min_{1\leq m\leq l+1}\left(C\left[i,j-m,k-m\right]+c\left(D\left(m\right)\right)\right) & \text{if}\;X\left[j\right]=Y\left[k\right]\\ +\infty & \text{otherwise} \end{array}\right)$$

where *l *is the largest value such that *X*[*j *− *l, j*] = *Y *[*k *− *l, k*] and *X*[*j *− *l, j*] has an occurrence in *A*.

5 [*a, x, −*]: Reversal in both *X *and *Y*, and loss in *Y*. (The case [*a, −, y*] is treated similarly)

$$R_{X/Y}\left(i,j,k\right)=\left(\begin{array}{cc} min_{m\in E}\left(C\left[i-m,j-m,k\right]+c\left(R\left(m\right)\right)+c\left(L\left(m\right)\right)\right) & \text{if}\;E\neq \emptyset \\ +\infty \;\;\; & \text{otherwise} \end{array}\right)$$

where *E *is the set {*e*_1_, *e*_2_, . . . , *e_l_*} of maximum cardinality such that *A*[*i−e_p_, i*] is the reverse of *X*[*j *− *e_p_, j*] for all 1 *≤ p ≤ l*.

6 [*−, x, y*]: Duplication in both *X *and *Y*, and reversal in *Y*.

$$DR_{X/Y}\left(i,j,k\right)=\left(\begin{array}{cc} min_{m\in E}\left(C\left[i,j-m,k-m\right]+c\left(D\left(m\right)\right)+c\left(R\left(m\right)\right)\right) & \text{if}\;E\neq \emptyset \\ +\infty & \text{otherwise} \end{array}\right)$$

where *E *is the set {*e*_1_, *e*_2_, . . . , *e_l_*} of maximum cardinality such that *X*[*j−e_p_, j*] is the reverse of *Y *[*k *− *e_p_, k*] for all 1 *≤ p ≤ l *and *X*[*j *− *e_p_, j*] has an occurrence in *A*.

(similar formulae for *DR_Y __/X _*(*i, j, k*))

7 [*a, −, a*]: Loss in *X*. (The case [*a, a, −*] is treated similarly)

$$L_{X}\left(i,j,k\right)=\left(\begin{array}{cc} min_{1\leq m\leq l+1}\left(C\left[i-m,j,k-m\right]+c\left(L\left(m\right)\right)\right) & \text{if}\;A\left[i\right]=Y\left[k\right]\\ +\infty & \text{otherwise} \end{array}\right)$$

where *A*[*i *− *l, i*] is the longest suffixe of *A*[1, *i*] such that *A*[*i *− *l, i*] = *Y *[*k *− *l, k*].

8 [*a, −, −*]: Loss in both *X *and *Y*.

*L_XY_*(*i, j, k*) = *min*_0*≤m≤i−*1_(*C*[*m, j, k*] + *c*(*L*(*i *− *m*)))

9 [*−, x, −*]: Duplication in *X*. (The case [*−, −, y*] is treated similarly)

$$D_{X}\left(i,j,k\right)=\left(\begin{array}{cc} min_{1\leq m\leq l+1}\left(C\left[i,j-m,k\right]+c\left(D\left(m\right)\right)\right) & \text{if}\;X\left[j\right]\text{ has an occurrence in }A,X\text{ or }Y\\ +\infty & \text{otherwise} \end{array}\right)$$

where *l *is the largest value such that *X*[*j *− *l, j*] has an occurrence in *A, X *or *Y*.

After computing all the values leading to *C*(*|A|, |X|, |Y |*), the labeled alignment $L\left(Ā,\bar{X},Ȳ\right)$ obtained by a backtracking approach is not necessarily a feasible alignment as it may contain cycles. Notice that, since *A *is an ancestor of both *X *and *Y*, the target of an event cannot belong to *A*. Therefore only events with source and target in *X *or *Y *may belong to a cycle.

• STEP 2. RESOLVING CYCLES.

Let $O_{c}=\left\{O_{1},O_{2},\ldots ,O_{h}\right\}$ be a cycle of a labeled alignment $L\left(Ā,\bar{X},Ȳ\right)$ output by the above algorithm.

**Lemma 1 ***Any event of $O_{c}$ is a duplication event*.

*Proof: *Suppose the contrary and let *O_p _*be an event which is not a duplication. Then, by definition, the target $O_{p}^{T}$ of *O_p _*overlaps the source of $O_{p+1}^{S}$ of *O*_*p*+1_. Clearly, *O_p _*cannot be a loss as otherwise $O_{p}^{T}$ is empty and cannot have a non-empty intersection with $O_{p+1}^{S}$. Therefore *O_p _*should be a reversal. Assume w.l.o.g. that $O_{p}^{T}$ is in *Y *and let *Y*[*q*] be an element of both $O_{p}^{T}$ and $O_{p+1}^{S}$. Let *X*[*r*] be the character of *X *aligned with *Y*[*r*] in $L\left(Ā,\bar{X},Ȳ\right)$. Then *X*[*r*] should be in the source of *O_p _*and in the target of *O*_*p*+1_. But this leads to an interpretation of the corresponding column of $L\left(Ā,\bar{X},Ȳ\right)$ with two events instead of one, which is in contradiction with the recurrences leading to a minimum number of events for each column.   □

**Figure 2:**
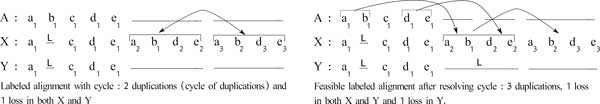
**Two different labeling for the alignment of strings *A*="abcde", *X*="acdeabdeabde" and *Y*="acde"**. Losses are denoted by "L" and duplications by arrows from source (indicated by bracket) to target. In the left labeling, "*a*_2_*b*_1_*d*_2_*e*_2_" is interpreted as the target of a duplication. In the right one, it is interpreted in $L\left(\bar{X},Ȳ\right)$ as a loss, and in $L\left(Ā,\bar{X}\right)$ as 2 duplications.

We resolve cycles as follows. Let  Z be the set of all overlapping strings {*Z*_1_, *Z*_2_, . . . , *Z_h_*} of $O_{c}$. Let $\varepsilon_{i}=\left\{z_{i_{1}},z_{i_{2}},\cdots z_{i_{l}}\right\}$ be a set of substrings of *Z*_*i*(1*≤i≤h*_) of minimum cardinality such that $z_{i_{1}}z_{i_{1}}\cdots z_{i_{l}}=Z_{i}$ and $z_{i_{k}\left(1\leq k\leq l\right)}$ has an occurrence in *A*. Let *Z_t _*be the string for which $|E_{t}|=min\left(\left|E_{1}|,|E_{2}|,\ldots |E_{h}\right|\right)$. Assume w.l.o.g. that *Z_t _*is a substring of *X*. Then *Z_t _*in $L\left(\bar{X},Ȳ\right)$ is covered by a loss in *Y*, and each substring of *Z_t _*in $L\left(Ā,\bar{X}\right)$ is covered by a duplication in *X *(source in *A*) (see Figure 2 for details).

*Complexity: *For simplicity, assume that *|A| *= *|X| *= *|Y| *= *n*. From the recurrences detailed above, each *C*(*i, j, k*) can be computed in linear time, leading to an *O*(*n*^4^) worst-time complexity for Step 1. Now, the complexity of Step 2 depends on the complexity for finding all cycles and resolving them. As cycles can only involve strings from *X *and *Y*, the problem reduces to the case of cycle-resolution for a pair-wise alignment, which has been shown quadratic (submitted journal version of [17]). This leads to a worst-time complexity of *O*(*n*^4^) for the whole algorithm.

## Experimental results

We call multiOrthoAlign our algorithm for the phylogenetic alignment problem based on the steinerization approach described in Section and using our 3-star algorithm for the iteration step.

In this section, we compare multiOrthoAlign with DupLoCut [20], on simulated and real-world instances. DupLoCut is an "almost" exact heuristic based on linear programming. For the sake of comparison with DupLoCut [20], we consider a model restricted to duplications and single gene losses. Indeed, DupLoCut is restricted to this evolutionary model. Moreover, we consider the default cost of one for each event.

### Simulations

We generate phylogenetic trees with 3 extant genomes. The genome at the root is generated in 2 steps. First, a random sequence R of length *n *on an alphabet of size *σ *is generated. Then, *l *moves (duplications and single gene losses) are applied to *R *where duplication length follows the geometric distribution of parameter 0.5. All other genomes along the tree are generated by applying *l *moves to their direct ancestor.

*Execution time: *We compare the running-time of our 3-star algorithm with that used in DupLoCut for the reoptimization steps. Running times were recorded using a 8-core Intel(R) 3.6 GHZ processor, with 16 GiB of memory. Table 1 gives average running times after one round (iteration) of reoptimization for simulations generated with three choices of parameters *n, σ *and *l*. Although multiOrthoAlign's running time increases slightly with increasing values of *n, σ *and *l*, it is still within a few minutes for *n *= 250. In comparison, the same data took more than 6 hours to be processed by DupLoCut.

**Table 1:** Average running times in minutes after one round of reoptimization

Parameters (n, *σ *= n/10, l = n/3)	multiOrthoAlign	DupLoCut
(150,15,50)	0.33	48.93
(200,20,67)	0.90	110.12 (≃ 2 hours)
(250,25,84)	3.07	370.60 (> 6 hours)

Running times comparison between multiOrthoAlign and DupLoCut on simulated triplet phylogenies after one round of reoptimization. Times are averaged over 50 simulations for the first choice, and 10 simulations for the second and the last one. Average running times are reported in minutes.

*Accuracy: *In order to test the performance of multiOrthoAlign in terms of accuracy, we used two measures: $Error=\frac{Inf-Opt}{Inf}$ where *Inf *is the number of events inferred by multiOrthoAlign and *Opt *is the "almost optimal" number of events obtained by running DupLoCut; $Accuracy=\frac{NbOpt}{Total}$, where *N bOpt *is the number of simulations among *Total *(number of all simulations) for which multiOrthoAlign returns the same number of events as DupLoCut.

**Figure 3:**
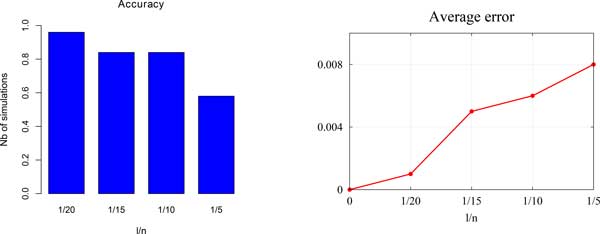
**Performance of multiOrthoAlign in terms of accuracy**. The genome length is fixed to *n *= 100 and the alphabet size to *σ = n/*2; diagrams are obtained by varying the number of moves *l *(x-axis is *l/n*); results are averaged over 50 simulations. Left: Accuracy of multiOrthoAlign compared with DupLoCut. Right: the average *Error*.

The same algorithm (2-SPP [19]) was used for the initialization step of both multiOrthoAlign and DupLoCut. Figure 3 gives results for different choices of the parameter *l*. With ratios *σ/n *= 1*/*2 and *l/n *= 1*/*20, multiOrthoAlign returns the same cost as DupLoCut for more than 96% of the simulations. This accuracy rate remains stable for decreasing alphabet size (results not shown), i.e., for increasing number of gene copies, but decreases quickly as the number *l *of moves increases (left diagram of Figure 3). However, the average *Error *remains lower than 0.008 (right diagram of Figure 3).

**Figure 4:**
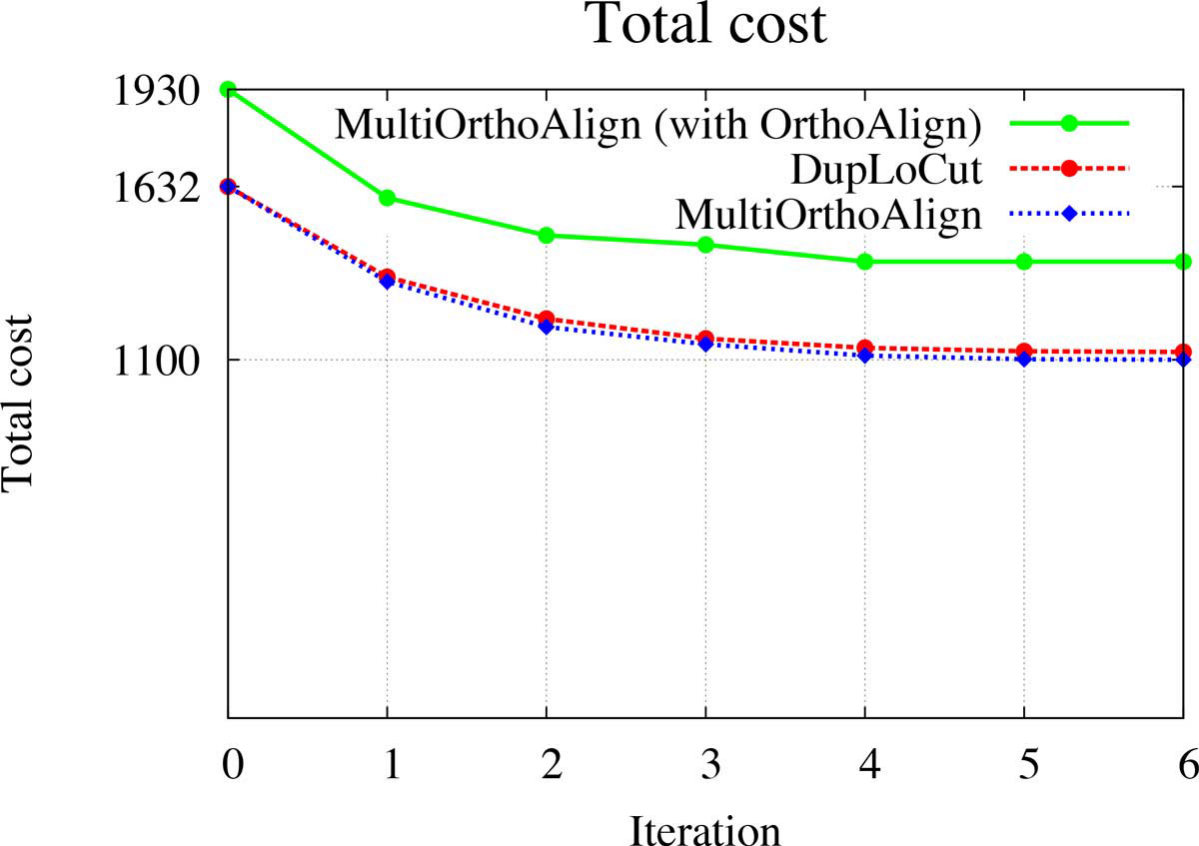
**Total cost obtained by **multiOrthoAlign **versus the one obtained by DupLoCut**. Blue refers to the cost obtained by multiOrthoAlign when we used the 2-SPP algorithm for the initialization step, green to the cost obtained by multiOrthoAlign when we used OrthoAlign for the initialization step, and red to the cost obtained by DupLoCut.

In order to test the algorithm on larger trees, we generated a phylogenetic tree with 100 extant genomes. The genomes along the tree were generated as described above for triplet phylogenies, with parameters *n *= 100, *σ *= 50 and *l *= 5. Figure 4 illustrates the total cost of the tree (number of duplication/single gene loss events) obtained after each iteration of multiOrthoAlign (blue line) and DupLoCut (red line). After the initialization step (iteration 0), the total cost obtained by multiOrthoAlign is 1632. After 6 rounds of reoptimization, the two programs converge to a local minimum (no improvement can be made), with a total cost of 1100 for multiOrthoAlign and of 1124 for DupLoCut. Our cost is always slightly better in this case. Notice that, although DupLoCut is "almost" exact for the median problem, the whole steinerization procedure does not guarantee any optimality result.

Using OrthoAlign instead of the 2-SPP algorithm for the initialization step would be something natural to do for reducing the running time of the whole procedure. However, as illustrated in Figure 4 (green line), the initial assignment obtained with OrthoAlign in this case leads to a cost of 1930 which is far from the best solution found. Notice that 2-SPP is an exact algorithm for pair-wise alignment and OrthoAlign is a heuristic which does not guarantee the optimal result. multiOrthoAlign converge to a local minimum of 1401 events after 4 rounds of reoptimization.

### Real data

**Figure 5:**
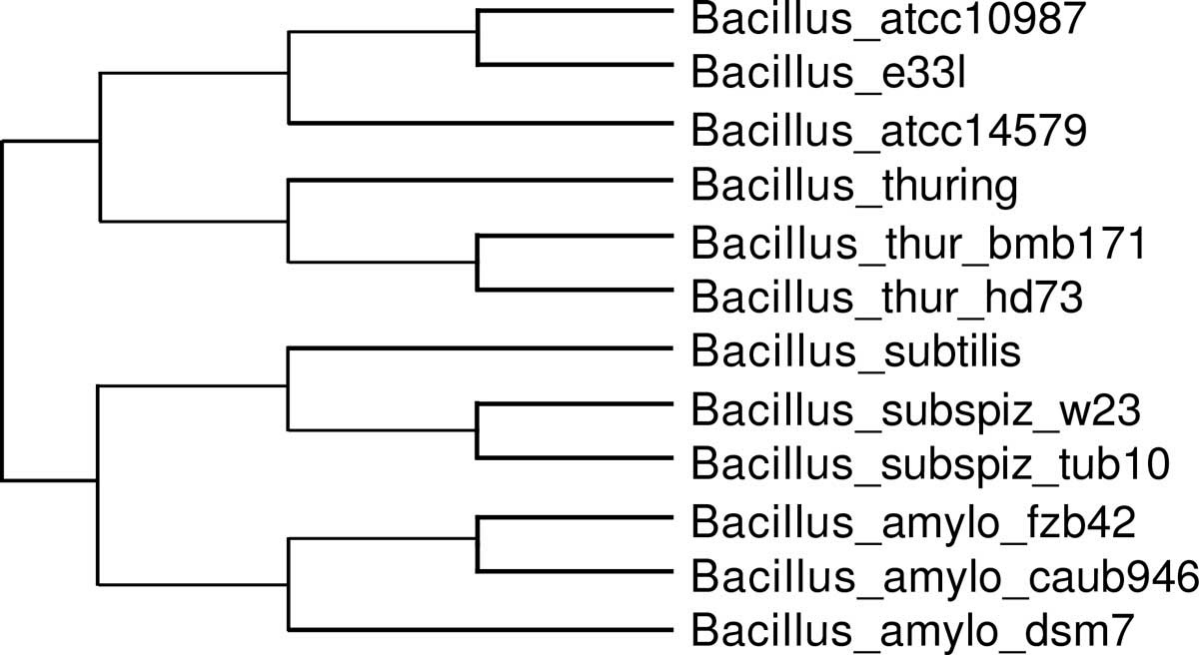
**Phylogenetic tree of the 12 Bacillus strains taken from the webpage (http://ccb.jhu.edu/software/duplocut)**.

We also compared the two approaches on the set of real-world instances used in [20]. The set contains the stable RNA genes of 12 *Bacillus *strains of four species (amyloliquefaciens, subtilis, thuringiensis, and cereus). The phylogeny shown in Figure 5 is taken from the webpage (http://ccb.jhu.edu/software/duplocut).

Using 2-SPP for the initialization step, multiOrthoAlign leads to a cost of 136 after the initialization step, and converges to a local minimum of 123 events after 2 rounds of reoptimization. As for DupLoCut, it converges to a local minimum of 120 events after 5 rounds of reoptimization. However, using OrthoAlign instead of 2-SPP for the initialization step, multiOrthoAlign leads to a cost of 131 after the initialization step, which is not refined by subsequent iterations. It therefore appears that 2-SPP is a more appropriate initialization procedure than OrthoAlign.

## Conclusion

We have developed multiOrthoAlign, a phylogenetic alignment algorithm for a genome-wide evolutionary model involving duplications, losses and rearrangements. It uses a generalization of OrthoAlign [21], a recently developed pair-wise alignment algorithm, to the median of three genomes. Our algorithm for the median problem is a heuristic that does not guarantee any optimality result. Compared with DupLoCut, the most closely related existing algorithm, multiOrthoAlign exhibits similar results but is much faster. The method can be easily extended to other contentmodifying and rearrangement operations such a substitutions, insertions, tandem duplications or inverted duplications. However, the more operations we add, the more challenging is the problem of finding appropriate costs for operations, and appropriate criteria to deal with the non-uniqueness of solutions.

## Competing interests

The authors declare that they have no competing interests.

## Declarations

Publication charges for this work was funded by The Natural Sciences and Engineering Research Council of Canada (NSERC).

This article has been published as part of *BMC Genomics *Volume 15 Supplement 6, 2014: Proceedings of the Twelfth Annual Research in Computational Molecular Biology (RECOMB) Satellite Workshop on Comparative Genomics. The full contents of the supplement are available online at http://www.biomedcentral.com/bmcgenomics/supplements/15/S6.
